# Maximizing the Clinical Benefit of Radiotherapy in Solitary Plasmacytoma: An International Multicenter Analysis

**DOI:** 10.3390/cancers12030676

**Published:** 2020-03-13

**Authors:** Khaled Elsayad, Michael Oertel, Laila König, Sebastian Hüske, Emmanuelle Le Ray, Mohamed A.M. Meheissen, Amr Abdelaziz Elsaid, Essam Elfaham, Jürgen Debus, Youlia Kirova, Klaus Herfarth, Hans Theodor Eich

**Affiliations:** 1 Department of Radiation Oncology, University Hospital of Munster, Albert-Schweitzer-Campus 1, Building A1, 48149 Munster, Germany; Michael.Oertel@ukmuenster.de (M.O.); hans.eich@ukmuenster.de (H.T.E.); 2 Department of Radiation Oncology, University Hospital Heidelberg, 69120 Heidelberg, Germany; Laila.Koenig@med.uni-heidelberg.de (L.K.); hueske@stud.uni-heidelberg.de (S.H.); juergen.debus@med.uni-heidelberg.de (J.D.); Klaus.Herfarth@med.uni-heidelberg.de (K.H.); 3 Department of Radiation Oncology, Institut Curie, 75248 Paris, France; emmanuelle.leray@gmail.com (E.L.R.); youlia.kirova@curie.fr (Y.K.); 4 Hematology Department, Hospital of Argenteuil, 95107 Argenteuil, France; 5 Department Alexandria clinical Oncology Department, Alexandria University, 21131 Alexandria, Egyptamrelsaid@yahoo.com (A.A.E.); 6 Specialized universal network of Oncology (SUN), 21648 Alexandria, Egypt; emf86@hotmail.com; 7 Versailles St Quentin University, 78000 St Quentin, France

**Keywords:** extramedullary, solitary bone plasmacytoma, combined radiotherapy, IMRT

## Abstract

*Objective*: Although local definitive radiotherapy (RT) is considered the standard of care for solitary plasmacytoma (SP), the optimal RT parameters for SP patients have not been defined. The aim of this retrospective study is to analyze the effectiveness of various RT doses, volumes, and techniques, as well as to define the relevant prognostic factors in SP. *Methods*: Between 2000 and 2019, 84 patients, including 54 with solitary bone plasmacytoma (SBP) and 30 with extramedullary plasmacytoma (EMP), underwent RT at six institutions. *Results*: The overall RT median dose was 42 Gy (range, 36.0–59.4). The median follow-up period was 46 months. Overall, the local control (LC) rate was 96%, while the complete remission (CR) rate was 46%. The 5-year local relapse-free survival (LRFS), multiple myeloma-free survival (MMFS), progression-free survival (PFS), and overall survival (OS) rates were 89%, 71%, 55%, and 93%, respectively. Using an RT dose above 40 Gy was associated with a higher complete remission (CR) rate and a lower rate of local relapse. Modern irradiation techniques were associated with a trend toward a higher LC rate (98% vs. 87% for conventional, *p* = 0.09) and a significantly lower local relapse rate (6% vs. 25% for conventional, *p* = 0.04). However, RT dose escalation and technique did not lead to a significant effect on MMFS, PFS, and OS. Univariate analyses identified several patient characteristics as potentially relevant prognostic factors. In SBP patients, systemic therapy administration was associated significantly with MMFS and PFS rates. *Conclusion*: Using an RT dose >40 Gy and modern RT techniques may improve the local control and reduce the rate of relapse, without a significant impact on survival rates. The addition of systemic therapies may improve the MMFS and PFS rates of SBP patients.

## 1. Objective

Solitary plasmacytoma (SP) is a rare plasma cell disorder characterized by localized lesions and the secretion of a monoclonal immunoglobulin [1,2]. The major types of SP are solitary bone/intraosseous plasmacytoma (SBP) and extramedullary/extraosseous plasmacytoma (EMP). Approximately two-thirds of cases are SBP, predominantly affecting the axial skeleton, while EMP is frequently found in the head and neck mucosa [2,3,4].

The diagnosis of SP is based on the exclusion of systemic involvement and an absence of end-organ damage attributable to plasmacytoma (e.g., anemia, hypercalcemia, renal insufficiency, or multiple lytic lesions) [5]. Imaging examinations, such as full-body magnetic resonance imaging (MRI) or positron emission tomography (PET)-computed tomography (CT), are required for the initial diagnosis of SP, as well as to monitor the therapeutic response to, and disease progression of, multiple myeloma (MM) [2,6,7]. 

SP can be treated effectively with local radiotherapy (RT) with or without surgical resection, using a local control (LC) rate as high as 95%–100% [3,7,8,9,10]. However, because of the relatively small number of patients with SP and the lack of randomized clinical trials, there is not yet a consensus on the optimal RT parameters (i.e., dose, volume, and technique) for patients with SP [2]. Despite the LC that is achieved following RT, nearly half of the patients with SBP and nearly one-third of patients with EMP develop new bone lesions and experience a progression to MM [7]. With the goal of enhancing survival rates and delaying disease progression, several investigators have combined RT with systemic treatments [11,12,13,14,15]. Recently, a new classification of SP associated with a high-risk of disease progression was defined by the criteria of minimal marrow involvement and <10% clonal bone marrow plasma cells [1,5,7,16,17]. 

Therapy de-escalation has been gaining interest for various hematologic malignancies [18,19,20,21,22]. Therefore, we undertook an international multicenter analysis to investigate the impact of different RT parameters on patient outcomes and to identify clinically relevant prognostic factors of SP that have not yet been defined. 

## 2. Materials and Methods 

The present retrospective study involved a cohort of 84 patients (29 females, 55 males) with SP, who each underwent an RT course between January 2000 and October 2019 at one of six closely cooperating institutions (Table S1). This study was approved by the ethics committees of all participating centers. The median age of the patients was 57 years (range, 22–81). The median RT dose given was 42 Gy (range, 36.0–59.4). There were 30 (36%) patients with solitary EMP and 54 (64%) with SBP manifestations. All lesions were examined histologically and diagnosed in accordance with the international myeloma working group criteria ^5^. All patients underwent CT scans. Additional examinations using MRI (*n* = 41) or PET-CT (*n* = 5) were performed in 46 patients (55%). The median tumor maximum diameter was 4 cm (range, 0.7–9.6). Two-thirds of the lesions (69%) were ≤5 cm in maximum diameter. The most common sites were the head and neck (*n* = 29) and the thoracic region (*n* = 28). Among patients with SBP, the most common site was the spinal column (*n* = 20/54, 37%) followed by the pelvis (*n* = 15/54, 28%). Among patients with EMP, the most common site was the upper aerodigestive mucosal surfaces (*n* = 23/30, 77%) followed by the urogenital system tract (*n* = 5/30, 17%). Only 3/30 patients with EMP (10%) presented bone infiltration and erosion. Serological profiles, including the following tests, were obtained before RT for 80 patients: hemoglobin level, serum calcium, serum protein, immunofixation (urine and serum), β2-microglobulin, and lactate dehydrogenase (Table 1). 

The planning target volume was produced by adding a 1–3 cm margin to the gross tumor volume or clinical target volume (based on the postoperative tumor bed). The clinical target volume was determined based on imaging information from CT, MRI, and/or PET-CT scans. Patients were divided into low-dose RT (ldRT; 36–40 Gy), standard-dose RT (sdRT; >40–50 Gy), and high-dose RT (hdRT; >50 Gy) groups. 

## 3. Statistical Analysis

All statistical analyses were conducted with SPSS (version 25.0) software. Differences were considered statistically significant at a *p*-value ≤ 0.05. Fisher’s exact or Chi-squared tests were performed to analyze the relationships between pairs of categorical variables. A two-sample U test was used to study the relationship between a categorical variable and a continuous variable. The local relapse-free survival (LRFS) was calculated from the initiation of RT until the time of the documented local plasmacytoma relapse. The multiple myeloma-free survival (MMFS) was calculated from RT initiation until the progression to MM. Progression-free survival (PFS) was calculated from the initiation of RT until the time of the documented local relapse or progression to MM or death. Overall survival (OS) was calculated from the first day of RT. The median time periods are reported with inter-quartile ranges (IQRs). Time-dependent Kaplan–Meier event curves were generated and compared with a log-rank test. Variables shown by a univariate analysis (*p* ≤ 0.1) to be associated with LRFS, MMFS, PFS, or OS were entered into a Cox proportional hazard regression model with a stepwise backward selection for multivariate analysis; the regression model results are reported as hazard ratios (HRs).

### Definition of Response

The treatment response was assessed at a 2- to 3-month follow-up appointment with a clinical examination and radiology (CT or MRI). Complete remission (CR) was defined as a CR of plasmacytoma, while a partial response (PR) was represented by any sub-CR response exceeding 50% regression. Local progression was defined as a >25% expansion of plasmacytoma lesions. Otherwise, the ongoing presence of lesions was classified as a stable disease (SD). In the CT scans, SBP patients with unchanged or residual sclerosis of lytic lesions, in the absence of a PET follow-up, were classified as having SD.

## 4. Results 

### 4.1. Treatments Administered

Of the 84 patients in the study, 44 (53%) received ldRT (median dose, 40 Gy), 22 (26%) received sdRT (median dose, 48 Gy), and 18 (21%) were treated with hdRT (median dose, 54 Gy). Additionally, elective nodal irradiation was applied to 6/84 patients (7%). Modern radiation techniques were employed for 68 patients [81%; 64 intensity-modulated RT (IMRT) and four proton therapy] (Table 2). The median RT dose for patients who received IMRT, proton therapy, or 3D-CRT were 41 Gy (range, 36–59,4), 52 Gy (range, 42–54), and 40 Gy (range, 36–50), respectively. Resection operations were performed on 20/84 patients (24%; 18 subtotal and 2 gross total). Twenty-eight patients (33%) received systemic therapies prior to (*n* = 5) or adjuvant to (*n* = 23) their RT courses, including 24 SBP patients and only 4 EMP patients (*p =* 0.004). Many more patients in the ldRT group (23/44, 52%) received systemic treatment than those in the sdRT (4/22, 18%) and hdRT (1/18, 6%) groups (both, *p* < 0.001). The most commonly applied systemic therapies were combined bortezomib/dexamethasone therapy (7/28; 25%) and combined lenalidomide/dexamethasone therapy (7/28, 25%). The median follow-up period was 46 months (range, 2–154). Data collection continued until the last visit (*n* = 72) or death (*n* = 5). Only seven patients lost to follow-up after a median period of 4 months (range, 2–21; IQR, 11), with at least one post-treatment examination.

### 4.2. Response Rates

The LC rate for the whole cohort was 96% (46% CR, 10% PR, and 40% SD), with no significant effect of lesion diameter (*p* = 0.7) or EMP versus SBP diagnosis group (*p* = 0.7) on the LC rate. Based on the follow-up CT scans, we found that the CR rate was significantly higher among EMP patients (84%) than among SBP patients (26%, *p* < 0.001). The SD rates were significantly higher among SBP patients (63%) than among EMP patients (0%, *p* < 0.001). The overall response rates for EMP patients treated with ldRT, hdRT, and sdRT (and their regimens) were similar at 95%, 95%, and 100% (*p* = 0.60), respectively, whereas the CR rates differed among the dose regimen groups at 27%, 68%, and 67%, respectively (*p* = 0.001). The overall response rate was higher among patients who were treated with a modern RT technique (IMRT or proton therapy) than among patients treated with conventional techniques (98% vs. 87%, *p* = 0.09). 

### 4.3. Survival Rates 

**Figure 1:**
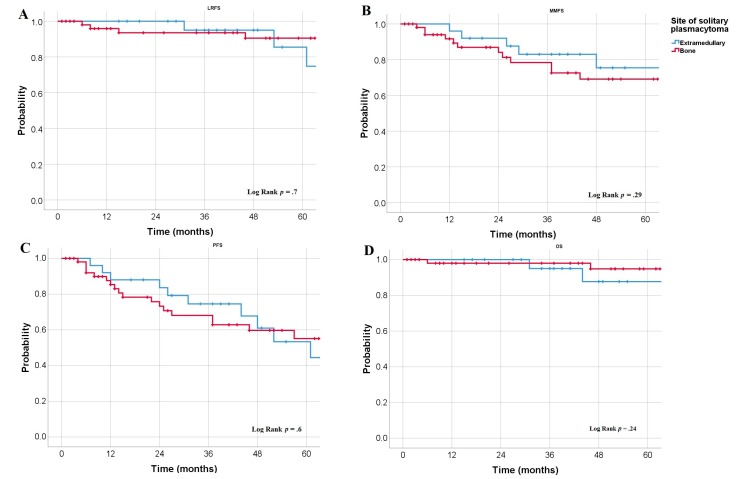
Kaplan–Meier curve derived estimates of LRFS (**A**), MMFS (**B**), PFS (**C**), and OS (**D**) according to the lesion site.

In the whole cohort, the 3-year LRFS rate was 94%, and the 5-year LRFS rate was 89% (median LRFS rates were not reached). The 3 year and 5 year LRFS rates did not differ significantly (*p* = 0.7; Figure 1A) between the SBP patients (93% and 90%, respectively) and EMP patients (95% and 86%, respectively). Overall, the median MMFS rate was 95 months, with a 3-year MMFS rate of 80% and a 5-year MMFS rate of 71%. The median MMFS rates were similar among patients with EMP (not reached) compared to patients with SBP (95 months, *p* = 0.29; Figure 1B). The 3-year MMFS rates were 83% for the EMP group versus 78% for the SBP group, whereas the 5-year MMFS rates were 76% for the EMP group versus 69% for the SBP group. In the whole cohort, the median PFS rate was 73 months with a 3-year PFS of 70% and a 5-year PFS rate of 55%. The median PFS rates were similar (*p* = 0.6; Figure 1C) between the SBP (3 year, 74%; 5 year, 53%) and EMP (3 year, 68%; 5 year, 55%) groups. Overall, we observed a 3-year OS rate of 97% and a 5-year OS rate of 93%. The OS rates were similar (*p* = 0.24; Figure 1D) between SBP patients (3 year, 98%; 5 year, 95%) and EMP patients (3 year, 95%; 5 year, 88%). 

**Figure 2:**
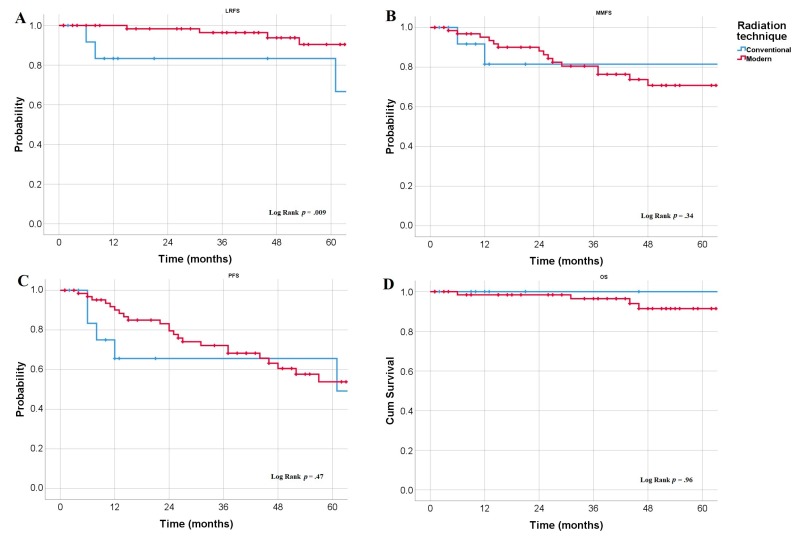
Kaplan–Meier estimates of LRFS (**A**), MMFS (**B**), PFS (**C**), and OS (**D**) according to radiation technique.

Regarding patient and treatment characteristics, a significantly greater 5-year LRFS rate was observed following modern RT techniques (90%) than following conventional techniques (83%, *p* = 0.009; Figure 2A), without a significant impact on MMFS, PFS, OS (Figure 2B–D). As well as higher LRFS among patients with normal protein levels compared with those with elevated protein levels (*p* < 0.001). We observed (non-significant) trends toward greater MMFS rates among patients with pharyngeal EMP lesions (*p* = 0.09 vs. non-pharyngeal EMP), patients younger than 60 years old (*p* = 0.07 vs. older patients), patients exhibiting a CR to RT (*p* = 0.08 vs. PR/SD), and patients with normal β2 microglobulin levels (*p =* 0.1 vs. elevated). In the subgroup analysis, we found that normal β2 microglobulin levels (*p =* 0.05) and being younger than 60 years old (*p* = 0.02) were significantly associated with greater MMFS rates among patients with SBP. Moreover, the prognostic benefits of systemic therapy (*p* = 0.009) and young age (*p* = 0.03) were observed selectively among SBP patients. In a subgroup analysis of patients treated with IMRT (*n* = 64), an RT dose escalation ≥50–59.4 Gy was associated with a significantly higher CR rate (74% vs. 10%, *p* < 0.001) and a trend toward higher MMFS (71% vs. 58%, *p* = 0.1). Patients younger than 60 years old (*p* = 0.008) had a significantly higher PFS rate than older patients. Meanwhile, we observed trends toward greater PFS rates in patients with non-spinal SBP (*p* = 0.09, vs. spinal SBP), patients with a normal β2 microglobulin level (*p* = 0.06), and patients who received systemic therapy (*p =* 0.1). In the follow-up subgroup analysis, the benefits of systemic therapy (*p* = 0.013), normal β2 microglobulin levels (*p* = 0.05), and being younger than 60 years old (*p* = 0.02) remained significant among patients with SBP. Patients with small lesions (maximum diameter <5 cm) showed a trend toward a higher OS rate (*p =* 0.07). We did not detect any significant LC or survival advantages in patients who underwent surgical resection prior to RT.

### 4.4. Relapse Pattern

Twenty-nine patients (35%) suffered a relapse within the study period, including 10% who had a local relapse and 25% whose disease progressed to overt MM. The pattern of relapse did not differ between the EMP and SBP patients. The median time to local progression was 38 months (IQR = 49), and the median time to MM development was 25 months (IQR = 22). We did not detect any difference between the EMP and SBP groups regarding time to relapse (*p* = 0.7), nor did we find a significant correlation between the plasmacytoma size and relapse rate (*p* = 0.7). For the whole cohort, the local relapse rates for the ldRT group (14%) did not differ significantly (*p* = 0.17) from those of the sdRT (5%) and hdRT (6%) groups. However, in the subgroup analysis, we found that, among SBP patients, there was a significantly higher relapse rate following ldRT (12%) than that after the administration of higher doses (0%, *p* = 0.04). No significant difference in the rate of progression to MM between different RT doses was found. Moreover, the local relapse rate was significantly lower among patients treated with modern RT techniques (6%) than among those treated with conventional techniques (25%, *p* = 0.04). One EMP patient who had tonsillar plasmacytoma experienced a cervical-node relapse and was treated successfully with RT. None of the patients who underwent elective nodal irradiation developed a nodal relapse. As most of the systemic treatments were performed in combination with low dose radiation in patients with SBP, we undertook a subgroup analysis after the exclusion of patients who received systemic therapy (*n* = 30). In this analysis, a trend toward higher CR patients who received more than 40 Gy (*n* = 10) was detected (40% vs. 20%, *p* = 0.06) compared to patients who received ≤40 Gy (*n* = 20). Moreover, 30% of lesions showed PR, and 30% showed SD, following a higher RT dose. In patients who received ≤40 Gy, the rates of PR and SD were 0% and 80%, respectively. However, there was no association between radiation dose and relapse rate or survival (*p* > 0.05). Interestingly, the four EMP patients treated with proton therapy had a complete response after RT, with a median survival of 34 months (range, 20–48; 95%-CI: 14–54). Only one patient (25%) developed MM after 24 months.

### 4.5. Toxicities 

RT was well tolerated, with none of our patients experiencing a severe (grade 3 or 4) adverse event (AE). During and within 6 months following the RT courses, almost half of the patients showed grade 1 AEs (47%), 22% of patients experienced grade 2 AEs, and 8% had grade 3 AEs. The rates of late grade 1, 2, and 3 AEs (>6 months after RT) were 33%, 7%, and 2%, respectively. The most common acute AEs were erythema, mucositis, and local pain. The most common late toxicity symptoms were xerostomia and fatigue. There were no radiation breaks. Relative to the use of conventional RT, the use of a modern RT technique, such as IMRT or proton therapy, was not associated with significant differences in the rates of moderate (grade 2/3) acute AEs (29% vs. conventional 33%, *p* = 0.7) or late AEs (7% vs. conventional 17%, *p* = 0.4). The rates of acute AEs (*p* = 0.08) and late AEs (*p* = 0.4) did not differ significantly between the different RT-dose groups.

### 4.6. Cox proportional Hazard Model

Our univariate analysis results (Table 3) suggest that age, RT technique, and serum protein level may impact the LRFS rate, whereas CR from RT, β2 microglobulin levels, and hemoglobin levels may be predictors of the MMFS rate, and the PFS rate may be related to age, β2 microglobulin level, serum protein level, hemoglobin level, and administration of systemic therapy. Our subgroup analysis revealed a significant impact of systemic therapy on the MMFS rate (HR = 0.109, *p* = 0.03) and PFS rate (HR = 0.239, *p* = 0.02) in SBP patients only. Only age and tumor diameter were found to be potentially associated with OS. 

## 5. Discussion 

Several studies have reported high response rates with an RT dose of 40 Gy [2,11,23,24]. However, concerns have been raised regarding whether higher RT doses may be more advantageous in patients with large lesions [3,25,26,27,28]. In the present large international multicenter study of SP, although we did not find a significant difference in the overall response rate in relation to radiation dose, we did find a significantly improved CR rate following an RT dose of >40 Gy (68% in sdRT group and 67% in hdRT group) compared to that observed with ≤40 Gy (27%), with no significant increase in the rate of toxicity under a higher radiation dose. However, there was no association found between RT dose and survival rates (MMFS, PFS, and OS). Notwithstanding, one-fourth of the patients in our cohort progressed to MM with no significant differences in the relapse rates between the different RT dose groups. On the other hand, several investigators failed to demonstrate an RT dose-response relationship in the SP treatment [13,29,30], and the International Lymphoma Radiation Oncology Group continues to recommend a dose of 40–50 Gy for patients with SP [2]. 

Although there has been a growing interest in modern RT techniques that can spare at-risk organs, such as IMRT and proton therapy, as potential curative treatments for hematological malignancies, studies evaluating the application of modern techniques among patients with SP are limited. In a study of 46 SP patients, Mignot et al. [11] reported that IMRT was well tolerated with no dermatitis of grade 2 or higher. Interestingly, the present comparisons of outcomes among patients treated with modern RT techniques compared to those treated with three-dimensional conformal RT (3D-CRT) suggested a potential trend toward higher overall response rates following IMRT and proton therapy, with a significant effect of technique on the LRFS rate found in our univariate and multivariate analyses. Moreover, the local relapse rate was significantly lower among patients who were treated with modern RT techniques than among those who were treated with 3D-CRT. However, this difference did not lead to a significant effect on MMFS, PFS, and OS. In accordance with the data for the head and neck malignancies, proton therapy and IMRT proved to optimize the dose, thereby sparing nearby organs in order to deliver high radiation doses and improve local control [31,32].

Various parameters have been debated as potential clinical risk factors for SP patients [7,29], including serum protein level, β2 microglobulin level, and hemoglobin, which appear to be associated with poorer LC and PFS rates in the present cohort. Genetic and molecular studies have suggested that immunoglobulin heavy-chain translocations, cyclin-D overexpression, and trisomies may be risk factors for the development of MM [7,33]. The present work is consistent with prior studies reporting a negative effect of older age (>60 years) and large SP on survival [10,25,26,28].

In our cohort, the respective 3 year and 5 year MM progression probabilities for EMP (17% and 24%) and SBP (22% and 31%) patients were relatively low compared to the previously reported probabilities for the transformation to overt MM in EMP (5 year, ~30%) and SBP (5 year, 51%–56%) patients [10,26,29]. However, caution is required when interpreting previous studies because of the retrospective nature by which patients were diagnosed using plain radiographs and bone marrow cytology only, which are associated with a high likelihood of understaging [4,7]. Similar to our data, recent studies have shown progression-to-MM probabilities in the range of 10%–18% [5,8,11,27]. 

Flow cytometry and immunohistochemistry may enable the early detection of monoclonal plasma cells in bone marrow. The detection of abnormal clonal bone marrow plasma cells could help identify patients who are at an elevated risk of progression to MM [16,17]. With the use of advanced diagnostic modalities, patients with true SP (SBP or EMP) have only a 10% risk of progression to overt MM within 3 years, whereas patients with clonal marrow infiltration are at a higher risk of progression to MM (60% in SBP patients and 20% in EMP patients) [7]. Therefore, the revised SP classification rubric distinguishes between true SP clonal accumulation and SP with minimal marrow involvement [4]. 

Currently, systemic therapies are not recommended because of their limited supporting studies. However, these therapies might be of particular benefit for patients with PET-positive residual or refractory disease after RT [7]. A few studies with small sample sizes (≤61 patients) have demonstrated the clinical benefits of adjuvant or maintenance conventional antimyeloma therapies after SP irradiation [12,13,14]. For example, Mheidy et al. [12] demonstrated a better PFS rate with adjuvant systemic therapy irrespective of patient age, although the benefits were more pronounced in patients under 60 years old who had a 5-year PFS rate of 89% (vs. 46% in older patients) with adjuvant systemic therapy. They confirmed that the PFS rate benefit was retained in a multivariate analysis. Interestingly, they observed a lower relapse rate in patients given adjuvant therapy with the proteasome inhibitor bortezomib (26%; 79% of their cohort) relative to those not given an adjuvant therapy (67%) [12].

Based on the data for smoldering myeloma, immunomodulatory therapy with lenalidomide plus dexamethasone delayed disease progression and improved patient survival with minimal toxicity [34]. Recently, Mignot et al. reported that lenalidomide plus dexamethasone therapy given with RT was associated with MMFS and PFS rate-benefits in SP patients, presumably by eradicating, or at least attenuating, subclinical disease [11]. Accordingly, a phase III randomized trial is exploring whether lenalidomide plus dexamethasone therapy after RT can improve PFS rates, compared with RT only, in a high-risk SBP population (ClinicalTrials.gov: NCT02544308). Another phase III prospective trial (ClinicalTrials.gov: NCT02516423) is examining whether the post-RT use of ixazomib citrate (a proteasome inhibitor), lenalidomide, or dexamethasone with zoledronic acid is beneficial for patients with SBP, relative to zoledronic acid alone. The results are eagerly awaited with the hope that they will guide the development of risk-adapted therapeutic and follow-up approaches for this rare hematological malignancy. The effectiveness of novel antimyeloma agents (e.g., new proteasome inhibitors like carfilzomib), monoclonal antibodies (e.g., daratumumab or elotuzumab), and other new approaches, such as CAR-T cell therapy, needs to be investigated in high-risk SP patients [4]. 

The limitations of the present study include the limitations inherent in retrospective analyses, the relatively small number of patients in our study, and the fact that comprehensive molecular, laboratory, and imaging (i.e., PET-CT) data were not available for all patients in our analysis. In the absence of randomized trials, our findings warrant further investigation with the aim of maximizing the clinical benefits of RT and supporting the development of risk-adapted treatment approaches for patients who may require combination therapies inclusive of antimyeloma agents. 

## 6. Conclusions

In this large international multicenter analysis, RT for SP was well tolerated and highly effective, with a 96% LC rate. Using an RT dose above 40 Gy and modern irradiation techniques may improve LC and reduce relapse rates. However, there is no significant impact on MMFS, PFS, and OS. SBP patients given systemic treatments after RT may have longer MMFS and PFS rates. Several clinical risk factors may influence SP patient outcomes. 

## Figures and Tables

**Table 1:** Patient characteristics (*n* = 84).

Characteristic	Nr. (% or Range)	Site of Solitary Plasmacytoma		
		Extramedullary	Bone	*p*-value
Patients	84	30 (36%)	54 (64%)	
Median Age	57 y (22–81)	53 (22–81)	60 (28–77)	0.2
Gender	M: 55 (65 %) F: 29 (35 %)	22 (73%) 8 (27%)	33 (61%) 21 (39%)	0.3
*Immunohistochemical analysis*				0.3
Kappa light chain restriction	25 (30%)	12 (40%)	13 (24%)	
Lambda light chain restriction	25 (30%)	10 (33%)	15 (28%)	
Both	2 (2%)	0 (0%)	2 (4%)	
*Maximum diameter*				0.16
In cm	4 (0.7–9.6)	3.6 (0.7–7.9)	4.1 (1.3–9.6)	
*Serum Beta-2-microglobulin*				0.1
Elevated	4 (5%)	1 (3%)	3 (6%)	
*Serum protein*				0.17
Elevated	7 (8%)	2 (7%)	5 (9%)	
Low	5 (6%)	3 (10%)	2 (4%)	
*Serum albumin*				0.02
g/dL	4.5 (2.6–5.4)	4.6 (3.6–5.4)	2.8 (2.6–3)	
*Hemoglobin*				0.06
g/dL	13.3 (9–16.6)	14.1 (9.6–16.6)	13.2 (9–14.8)	
*Serum protein immunofixation*				0.3
Positive	36 (43%)	11 (37%)	25 (46%)	
Negative	29 (34%)	13 (43%)	16 (30%)	
Unknown	19 (23%)	6 (20%)	13 (24%)	
*Serum LDH*				0.13
Elevated	5 (6%)	5 (17%)	0 (0%)	
*Bence-Jones proteinuria*				0.12
Yes	12 (14%)	6 (20%)	6 (11%)	
*Sites of RT*				<0.001
Head and neck	29 (34%)	24 (80%)	5 (9%)	
Thorax	28 (33%)	2 (7%)	26 (48%)	
Abdomen	2 (2%)	0	2 (4%)	
Pelvis	21 (25%)	4 (13%)	17 (32%)	
Extremities	4 (5%)	0	4 (7%)	

M, males; F, females.

**Table 2:** Treatment characteristics (*n* = 84).

Characteristic	Nr. (% or Range)	Site of Solitary Plasmacytoma		
		Extramedullary	Bone	*p*-value
*Type of surgery*				<0.001
Resection	20 (24%)	15 (50%)	5 (9%)	
Biopsy	64 (76%)	15 (50%)	49 (91%)	
*Systemic therapy*				0.004
Yes	28 (33%)	4 (13%)	24 (44%)	
No	56 (67%)	26 (87%)	30 (56%)	
*Timing of systemic therapy*				
Prior to RT alone	5/28 (18%)	2/4 (50%)	3/24 (12%)	<0.001
Adjuvant alone	21/28 (75%)	0/4 (0%)	21/24 (88%)	
Both	2 (7%)	2/4 (50%)	0/24 (0%)	
*Radiation parameters*				
Med. radiation dose (range), Gy	42 (30–59.4)	50 (36–59.4)	40 (30–59.4)	<0.001
Radiation doses				<0.001
≤40 Gy	44 (53%)	3 (10%)	41 (76%)	
>40–50 Gy	22 (26%)	16 (53%)	6 (11%)	
>50 Gy	18 (21%)	11 (37%)	7 (13%)	
Med. fraction dose (range), Gy	2 (1.8–3)	2 (1.8–3)	2 (1.8–2)	0.03
Follow-up, m	46 (2–154)	40 (2–154)	52 (2–149)	0.4
Follow-up, IQR	53	48	55	
*RT technique*				0.02
Proton therapy	4 (5%)	4 (13%)	0	
IMRT	64 (76%)	22 (74%)	42 (78%)	
3D-CRT	16 (19%)	4 (13%)	12 (22%)	
*Response to RT*				0.7
Yes	81 (96%)	29 (97%)	52 (96%)	
No	3 (4%)	1 (3%)	2 (4%)	
Type of response				<0.001
CR	39/81 (48%)	25/29 (86%)	14/52 (27%)	
PR	8/81 (10%)	4/29 (14%)	4/52 (8%)	
SD	34/81 (42%)	0	34/52 (65%)	
*Relapse*				0.7
Yes	29 (35%)	9 (30%)	20 (37%)	
No	55 (65%)	21 (70%)	34 (63%)	
Pattern of relapse				0.6
Local	8 (10%)	3/30 (10%)	5/54 (9%)	
Multiple myeloma	21 (25%)	6/30 (20%)	15/54 (28%)	

Med: median; m: month; Gy: gray; RT: radiotherapy; IQR: interquartile range; CRT: 3-dimention conventional radiotherapy; IMRT: intensity-modulated radiotherapy; CR: complete response; PR: partial response; SD: stable disease.

**Table 3:** Univariate and multivariate analyses for LRFS, MMFS, PFS, and OS (*n* = 84).

Risk Factor	LRFS		MMFS		PFS		OS	
	HR	*p*	HR	*p*	HR	*p*	HR	*p*
*Univariate model*								
Age (years)	1.06	0.09	1.005	0.8	1.034	0.06	1.085	0.08
EMP vs. SBP	1.26	0.7	0.602	0.3	0.82	0.6	2.85	0.3
Diameter (cm)	0.89	0.7	1.17	0.3	1.06	0.6	1.74	0.07
Bone erosion	4.24	0.15	0.302	0.26	0.99	0.9	0.71	0.8
Surgical resection	2.29	0.26	0.67	0.5	0.91	0.8	0.49	0.4
RT dose (>40 Gy vs. ≤40 Gy)	0.38	0.2	1.39	0.5	1.12	0.7	1.92	0.5
Modern RT technique	0.83	0.02	2.08	0.4	0.72	0.5	1.06	0.9
CR after RT	1.39	0.6	0.42	0.09	0.67	0.3	1.01	0.9
B2-microglobulin (normal)	6.0	0.2	3.22	0.1	0.31	0.08	0.285	0.3
Serum protein (normal)	0.024	0.002	0.94	0.9	0.24	0.02	28.1	0.7
Hemoglobin (g/dl)	1.32	0.5	0.67	0.006	0.68	0.004	0.91	0.7
Systemic therapy	1.09	0.9	0.44	0.19	0.47	0.1	0.03	0.5
*Multivariate model*								
Age (years)	-	-	-	-	-	-	1.14	0.1
Diameter	-	-	-	-	-	-	2.29	0.03
Modern RT technique	0.036	0.012	-	-	-	-	-	-
Serum protein (normal)	0.019	0.008	-	-	-	-	-	-
Hemoglobin (g/dl)	-	-	0.676	0.006	0.655	0.004		

LRFS, local relapse-free survival; MMFS, multiple myeloma-free survival; PFS, progression-free survival; OS, overall survival; HR, hazard ratio; EMP, solitary extramedullary plasmacytoma; SBP, solitary bone plasmacytoma; RT, radiotherapy; IMRT; intensity modulated radiotherapy; CR, complete remission.

## Supplementary Materials

The following are available online at https://www.mdpi.com/2072-6694/12/3/676/s1, Table S1: Participating international centers.
