# Supplementary material for: Maximizing the Clinical Benefit of Radiotherapy in Solitary Plasmacytoma: An International Multicenter Analysis
Source: Cancers (Basel). 2020 Mar 13;12(3):676. doi: 10.3390/cancers12030676 (PMC7139814; doi:10.3390/cancers12030676)
Supplement: Supplementary file 1 [file cancers-12-00676-s001.pdf]

Supplementary Materials

Maximizing the Clinical Benefit of Radiotherapy in Solitary Plasmacytoma: An International Multicenter Analysis

Khaled Elsayad, Michael Oertel, Laila König, Sebastian Hüske, Emmanuelle Le Ray, Mohamed A.M. Meheissen, Amr Abdelaziz Elsaid, Essam Elfaham, Jürgen Debus, Youlia Kirova, Klaus Herfarth and Hans Theodor Eich

Table S1. Participating international centers.

Center Number	Principal Investigator	Address	Patients (*n*)
1	Khaled Elsayad	Department of Radiation Oncology, University Hospital of Munster	31
2	Youlia Kirova	Department of Radiation Oncology, Institut Curie, Paris, France	28
3		Versailles St Quentin University, St Quentin, France	
4	Laila König	Department of Radiation Oncology, University Hospital Heidelberg, Heidelberg, Germany	15
5	Mohamed A.M. Meheissen	Department Alexandria clinical Oncology Department, Alexandria University, Alexandria, Egypt	10
6		Specialized universal network of Oncology (SUN), Alexandria, Egypt	

© 2020 by the authors. Licensee MDPI, Basel, Switzerland. This article is an open access article distributed under the terms and conditions of the Creative Commons Attribution (CC BY) license (http://creativecommons.org/licenses/by/4.0/).
